# Recent advances in oblique plane microscopy

**DOI:** 10.1515/nanoph-2023-0002

**Published:** 2023-04-20

**Authors:** Jeongmin Kim

**Affiliations:** Department of Applied Bioengineering, Graduate School of Convergence Science and Technology, Seoul National University, Seoul 08826, Republic of Korea; Research Institute for Convergence Science, Seoul National University, Seoul 08826, Republic of Korea

**Keywords:** fluorescence microscopy, lightsheet microscopy, oblique plane microscopy, remote focusing

## Abstract

Oblique plane microscopy (OPM) directly captures object information in a plane tilted from the focal plane of the objective lens without the need for slow z-stack acquisition. This unconventional widefield imaging approach is made possible by using a remote focusing principle that eliminates optical aberrations for object points beyond the focal plane. Together with oblique lightsheet illumination, OPM can make conventional lightsheet imaging fully compatible with standard biological specimens prepared on microscope slides. OPM is not only an excellent high-speed volumetric imaging platform by sweeping oblique lightsheet illumination without mechanically moving either the sample or objective lens in sample space, but also provides a solution for direct oblique plane imaging along any orientation of interest on the sample in a single shot. Since its first demonstration in 2008, OPM has continued to evolve into an advanced microscope platform for biological, medical, and materials science applications. In recent years, many technological advances have been made in OPM with the goal of super-resolution, fast volumetric imaging, and a large imaging field of view, etc. This review gives an overview of OPM’s working principle and imaging performance and introduces recent technical developments in OPM methods and applications. OPM has strong potential in a variety of research fields, including cellular and developmental biology, clinical diagnostics in histology and ophthalmology, flow cytometry, microfluidic devices, and soft materials.

## 1 Introduction

Optical microscopy is essential for non-invasive observation of micro- to nano-scale features of specimens, especially biological objects such as cells, tissues, and small animals. Conventional microscopes capture planar information of the specimen at the focal plane of the objective lens designed to satisfy Abbe’s sine condition for aplanatic imaging [1]. This means that only a portion of the object in the focal plane is imaged sharply without optical aberrations, while the rest outside the focal plane can contribute to image blur or the background of the image. Such image blur is avoided in optical sectioning-based microscopy methods, including confocal microscopy and selective plane illumination microscopy (SPIM, also known as lightsheet microscopy) [2]. On the other hand, oblique plane microscopy (OPM) [3, 4] captures images along a plane inclined with respect to the focal plane of the microscope objective. Since most objective lenses are not designed to be used in this way, one might think that this imaging method is not ideal and will give poor imaging results due to optical aberrations. However, by using remote focusing [5, 6] that eliminates the major component of aberrations from out-of-focus objects, high-quality imaging along the inclined plane can still be obtained. OPM, which uses oblique lightsheet illumination through the same objective lens for fluorescence detection, can be an alternative to conventional lightsheet microscopy that uses two objectives placed perpendicular to each other in sample space. Due to its single objective geometry, OPM is fully compatible with standard biological specimens prepared on microscope slides. More importantly, OPM can achieve three-dimensional (3D) imaging without mechanically stepping either the objective or specimen in sample space, making it suitable for real-time volumetric imaging without sample perturbation.

Direct two-dimensional (2D) imaging of OPM along an oblique plane can also be useful when the imaging plane of interest of an object is not precisely aligned or parallel to the objective’s focal plane. This angular mismatch can occur during sample preparation when it is difficult to orient the morphological structure of interest to the focal plane [7–9] or when the prepared sample is loaded at an angle into the microscope. Also, in living cells attached to the microscope cover glass, some biological processes, such as active transport and endocytosis, can occur in a plane orthogonal to the coverslip surface, which cannot be directly imaged unless specialized sample devices are used [10]. Another example is the transportation of a trapped particle immersed in a non-diffracting beam that extends axially through the focal plane of the imaging objective [11]. In these situations, OPM can directly visualize the cross-sections of interest without the slow conventional z-stack acquisition.

Since its first demonstration in 2008 [3], OPM-based microscopy methods have continued to evolve as advanced microscope platforms for a variety of imaging applications. In recent years, many technological advances have been made in OPM to improve imaging performance such as spatial resolution [12–14], 3D imaging speed [15–19], and imaging field of view (FOV) [20–24]. This review aims to provide a comprehensive overview and recent progress in OPM technology and applications.

## 2 Overview of OPM

### 2.1 Optical configuration

**Figure 1:**
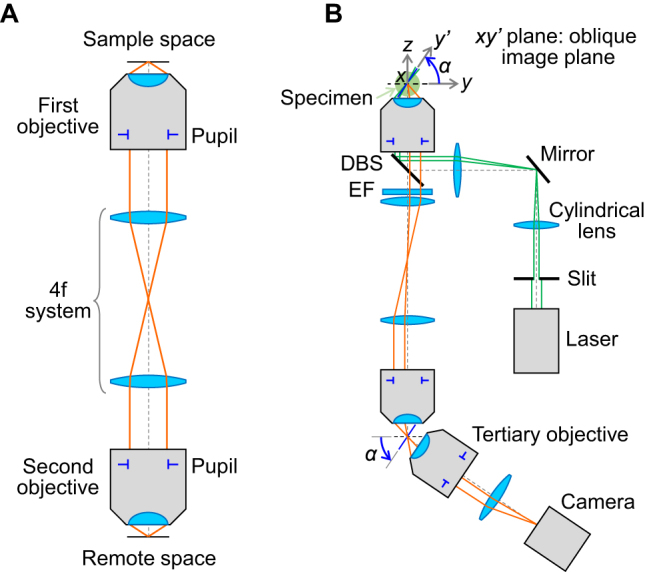
Schematic of oblique plane microscopy (OPM). (A) Optical remote focusing. (B) Widefield OPM with lightsheet illumination. DBS: dichroic beam splitter, EF: emission filter.

A key concept in OPM is optical remote focusing introduced by T. Wilson’s group in 2007 [5, 6], whereby an object forms its three-dimensional image in intermediate image space (or remote space) with an isotropic magnification of *n*_*o*_/*n*_*r*_, where *n*_*o*_ and *n*_*r*_ denote the refractive indices of object space and remote space, respectively. What is meant by isotropic here is that the lateral (*xy*) magnification is the same as the axial (*z*) magnification, so the formed 3D image has negligible dimensional distortion. This isotropic refocusing is achieved by connecting two objectives back-to-back through a 4f imaging system with a total magnification of *n*_*o*_/*n*_*r*_ as illustrated in Figure 1A, where the pupils of both objectives are optically conjugated. Then, due to the odd parity property of the first-order aberration function [6], the optical aberrations associated with capturing out-of-focus portions of the specimen with the first objective can be mostly canceled out by the second (or remote) objective. Therefore, 3D images can be formed in remote space without noticeable spherical aberrations even at higher numerical aperture (NA) refocusing.

Widefield OPM using remote focusing was proposed by C. Dunsby in 2008 [3], where a third microscope system is placed in remote space at an angle (*α*) to tilt the image plane as illustrated in Figure 1B. Together with lightsheet illumination along the oblique imaging plane (*xy'*) in the sample space, optically sectioned 2D fluorescence images are directly acquired in a single shot without beam scanning. Since the first objective is used here for both illumination and imaging, this layout is often referred to as a “single-objective” configuration, as opposed to conventional SPIMs [2] where two objectives in the sample space are placed perpendicular to each other for illumination and imaging, respectively. The widefield OPM was intended to be fully compatible with standard specimens prepared on microscope glass slides without the need for special sample holders for SPIM, while retaining the advantages of SPIM’s low photobleaching and phototoxicity [3]. 3D imaging was achieved by laterally stepping the specimen stage (in the *y* direction in Figure 1B) to obtain an oblique image stack that produces a parallelepiped-shaped image volume.

Widefield OPM requires careful optical design of the system layout depending mainly on the desired tilt angle (*α*). For small image tilts (*α* < 30°), lightsheet illumination may not be possible in the single-objective configuration even with a 1.2–1.49 NA objective, especially when the sheet thickness is thin. The smallest possible angle could be estimated for a given objective NA value as introduced in Appendix 2 of Ref. [25]. Thus, in this small angular range, non-confined epi-illumination without optical sectioning capability may be considered or the “dual-objective” configuration [26] for external lightsheet illumination should be applied. If the desired tilt angle is large (*α* > 60°; perhaps for deeper access into the specimen), mechanical interference can occur between two adjacent objectives in remote space. This is especially true for high resolution OPM systems with larger NA objectives which tend to have a shorter working distance with blunter housing of the front lens. A solution to the mechanical collision is to eliminate the need for the third (or tertiary) objective by placing a tilted mirror near the remote focus [8], creating a “single-objective” configuration in remote space. This layout allows adjustable imaging tilts of up to 90° without difficulty [27], at the expense of ∼50 % fluorescence light loss in the polarizing beam splitter [12, 28]. Here, the small mirror is set at half the desired tilting angle of the oblique section to rotate the oblique plane of interest back to the focal plane of the second objective for subsequent imaging. Whether to use two or three objectives in total, along with the choice of NA and magnification of each objective, also depends on other design specifications such as imaging FOV and spatial resolution.

### 2.2 Imaging resolution

Spatial resolution is one of the most important performance metrics in optical microscopy and is determined by the size of the point spread function (PSF) of an imaging system. Indeed, the Rayleigh criterion for two-point resolution in incoherent imaging is equal to the main lobe radius of the scalar paraxial PSF, 0.61*λ*/NA, in a circular aperture system, where *λ* denotes the wavelength of light used for imaging [29]. Spatial resolution can also be characterized in the spatial frequency domain by optical transfer function (OTF), an intrinsic property of imaging systems related to the Fourier transform of the intensity PSF [30]. In general, the higher the cutoff frequency of the OTF, the better the spatial resolution.

**Figure 2:**
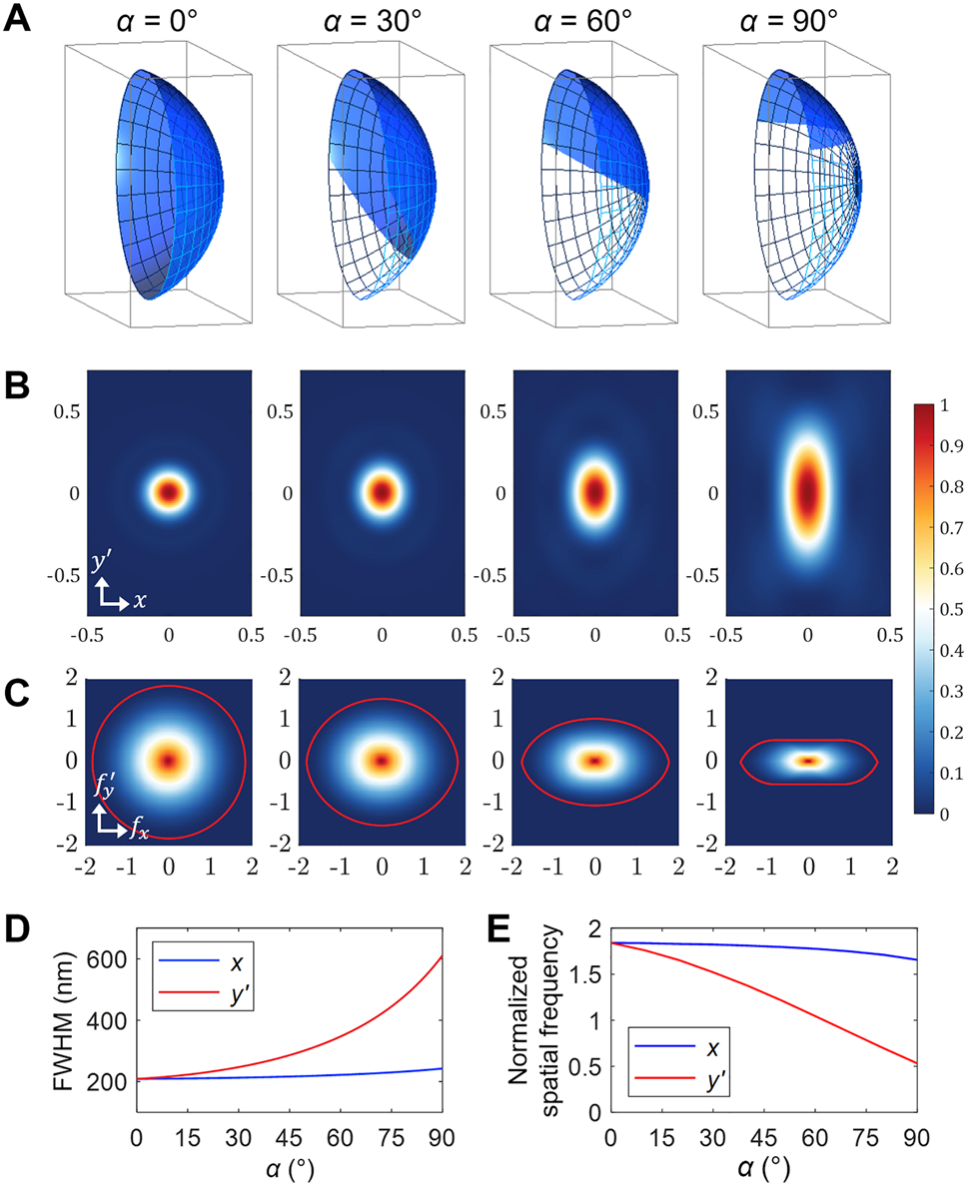
Anisotropic resolution in widefield OPM with 1.4 NA objectives (oil immersion). Adapted with permission from [31] © The Optical Society. (A) Shape of the effective 3D pupil (blue) at different angles (*α*). (B) Corresponding 2D vectorial PSF for *λ* = 519 nm (axis unit: μm). (C) Corresponding vectorial OTF at normalized spatial frequency. Red contour indicates the OTF passband. (D) Size of the PSF over *α*. (E) Change in the OTF cutoff frequency over *α*.

OPM exhibits anisotropic imaging resolution in the *x* and *y’* directions due to different PSF sizes. In the tilted geometry, the mechanical pupil of the tertiary objective can be underfilled with light for imaging. Thus, the effective pupil is no longer circularly symmetric, resulting in the anisotropic shape of the 2D PSF. In Ref. [3], the effective NA (NA_eff_) in the *y’* direction was calculated geometrically, from which the full width at half maximum (FWHM) of the PSF was estimated to be approximately 0.5*λ*/NA_eff_. Anisotropic resolution was discussed more qualitatively in Ref. [8], along with experimental characterization of resolution degradation for small imaging tilts (*α* = 0–14°). A more rigorous study based on vectorial diffraction theory was reported in Ref. [31], where 3D effective pupil functions were derived for *α* = 0–90° in the single-objective layout in remote space (Figure 2A). This pupil model was used to calculate the vectorial 2D PSF in which the polarization state of light and the apodization in the high NA objective were also considered. The calculation results clearly showed how objective NA and imaging angle affect the anisotropic resolution in widefield OPM (Figure 2B). In particular, the 2D PSF remains relatively circular when *α* < 30° but stretches significantly in the *y’* direction at higher tilt angles. For example, in axial plane imaging (*α* = 90°), the *y’*- and *x*-direction FWHM ratio of the PSF were estimated to be as large as 2.9 for an oil-immersion NA of 1.4. It was also shown that the 2D PSF is not simply the oblique slice of the conventional 3D PSF obtained from a circular effective pupil. In addition, the effective pupil in the *x* direction was found to slightly decrease at higher tilt angles, so the PSF size increases accordingly as shown in Figure 2D.

**Figure 3:**
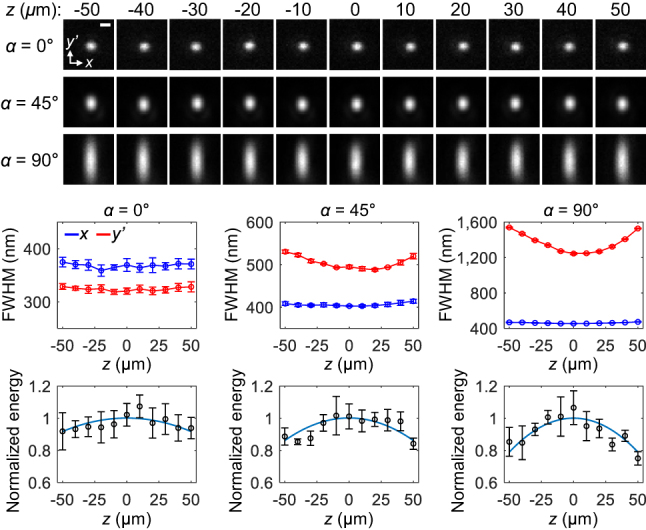
Axial DOF of OPM. Experimental PSF images of dark-red fluorescent beads at three different angles (1.2 NA, water immersion). Scale bar: 500 nm. Average FWHM and normalized energy of PSFs (mean ± s.d. for 9, 4 and 6 beads at *α* = 0, 45, and 90°, respectively). The sky-blue line represents the quadratic fit. Adapted from [12] © Springer Nature.

The vectorial formulation of 2D PSF in widefield OPM was further developed to reflect more generalized situations in Supplementary Information of Ref. [12]. They introduced an electric dipole to model a more realistic point source, considered polarizing optical elements such as polarizing beam splitters (PBSs) and quarter-wave plates during vectorial field tracing, and generalized the 3D pupil function when the remote objective has a larger cone angle than the first objective to minimize pupil loss. This improved PSF modeling provides excellent physical insight into the resolution of widefield OPM. In addition to the PSF, the vectorial OTF of widefield OPM was calculated from the fast Fourier transform of the 2D PSF as shown in Figure 2C [31]. They numerically computed the passband of the 2D OTF and analyzed the decrease of the cutoff frequency in the *x* and *y’* directions as *α* increases (Figure 2E). Their findings help to provide additional perspectives for frequency domain interpretation of the imaging resolution in widefield OPM.

### 2.3 Axial depth of field (DOF)

The dominant term for on-axis residual aberrations in remote focusing is proportional to *z* ^2^ sin^2^ *θ*, as derived as Eq. (18) in Ref. [6] or Eq. (3.13) in Ref. [32], where *θ* is the half-cone angle of the first objective and *z* is the defocus, i.e., the axial distance from the imaging plane to the focal plane of the first objective. Thus, this quadratic dependence of the aberrations on defocus limits the axial DOF, especially when high cone-angle objectives are used. The Strehl ratio (*S*), which represents the peak intensity ratio of aberrated and unaberrated PSFs, associated with the remote focusing was derived analytically using paraxial scalar diffraction theory as Eq. (23) in Ref. [6]. It could also be derived based on the scalar Debye approximation as Eqs. (3.14) and (3.15) in Ref. [32] that may be more accurate when using high NA objectives. The axial DOF could be defined as an axial range with *S* > 0.8 which is the conventional criterion for the diffraction limit. We note that the equations derived above are all for on-axis field positions, and the axial DOF may be further reduced for field positions away from the optical axis due to off-axis residual aberrations.

As an example, if two Olympus 60×/1.2 water-immersion objectives are used for remote focusing, the axial DOF is estimated to be as large as ±68 μm at *λ* = 685 nm and the peak intensity of the PSF theoretically drops 6 % at ±50 μm depth. This prediction from Eq. (23) in Ref. [6] agrees well with experimental measurements of PSF using fluorescent beads at *α* = 0° (Figure 3), where the PSF energy decreased by 8 % at ±50 μm depth with a relatively uniform PSF size. These results indicate good optical alignment with negligible residual aberrations through the imaging depth, although the Strehl ratio was not measured as in Ref. [8]. When the remote mirror was inclined from 0° to 45° and 90°, the PSF energy decreased further at ±50 μm depth by 14 % and 21 %, respectively, as shown in Figure 3. Also, the PSF size in the *y’* direction increased by ∼8 % and ∼25 % for *α* = 45° and 90°, respectively. Such PSF dimming and broadening in widefield OPM would give an axial DOF narrower than when *α* = 0°. The observed PSF degradation, especially at higher *α*, may be caused by asymmetric amplitude/phase modifications of light after Fresnel reflections from the tilted remote mirror [33] and/or possible vignetting-like energy loss for larger defocus, but it has not yet been fully investigated. In addition to the Strehl ratio, the change in PSF size through axial depth could be another figure of merit for setting the axial DOF.

More rigorous PSF and DOF estimates could be obtained through ray tracing simulations in commercial optical design software such as Zemax. In fact, PSF and spherical aberrations were simulated in remote focusing microscopy with a “realistic objective model” [34]. Yang et al*.* [18] simulated the Huygens PSF of widefield OPM in Zemax, where the undisclosed objective lens data was inferred from Nikon patents. These approaches would be useful for quantifying optical aberrations as a function of 3D field position, and they investigated the spatial variation of the PSF for multiple field positions within the imaging volume of interest. The simulated PSF will be even more accurate if the software supports calculating the vectorial PSF using a point source model at a specific polarization state.

### 2.4 Optical construction, alignment, and calibration

Widefield OPM is often built standalone without the use of a commercial microscope body [12, 17, 18, 35]. Kumar et al. [35] compared some of the latest OPM configurations and introduced an open access lightsheet OPM platform with details on how to design and build an OPM system. The most critical aspect of OPM design to ensure aberration-free imaging is precisely conjugating the pupils of the first and second objectives through the 4f imaging system. This optical conjugation is not optimally realized with the tube lens placed inside the microscope body because the distance between the first objective and the tube lens often falls outside the 4f condition [6]. Therefore, great care must be taken when attempting to integrate OPM into a commercial microscope without compromising imaging performance [36, 37]. Also, it is recommended to configure the remote focusing using “internally corrected” objectives [6] that do not require pairing with specific tube lenses for partial correction of aberrations such as lateral chromatic aberrations.

Alignment procedures for single-objective OPM are described in detail in several references including Refs. [12, 18, 38]. Fluorescent beads and/or grid test samples were used for precise pupil conjugation between the first and second objectives. In Supplementary Figure 4 of Ref. [12], the intensity and shape of PSF images of multicolor beads were examined across the imaging depth (∼100 μm) for *α* = 0°, from which the lateral misplacement (or decenter) of the second objective relative to the first objective was estimated and completely removed. For large lateral misplacement, coma aberrations become more pronounced in the PSF shape as the bead moves away from the nominal focal plane of the first objective. Then, by analyzing the lateral magnification from the bead images as a function of imaging depth for two emission wavelengths at *α* = 0°, they made the 3D magnification as uniform as possible by adjusting the distance between the second objective and the tube lens (Supplementary Figure 9 in Ref. [12]). Residual non-uniformity in 3D magnification for each color channel was corrected in the image data for correct pixel registration. Since the tilt angle (*α*) can be easily adjusted on the single-objective layout in remote space (with no third objective lens), such precise alignment is simply achieved at *α* = 0° and then *α* can be tuned as desired for OPM. In the now more widely used OPM geometry with two objectives in remote space, it is highly recommended to first align the entire tertiary imaging system to *α* = 0° for the pupil conjugation testing (inconvenient but necessary for high-quality imaging performance) and then to the desired angle. Optical conjugation of the objective pupils for uniform imaging magnification can also be achieved using grid test samples [18, 38] or 3D-fabricated calibration specimens [39].

## 3 Technical development and applications

The technology development and application of OPM has recently become much more active and exciting. Various OPM studies have been reported to improve imaging performance and expand their application fields, which are reviewed in the following four subsections.

### 3.1 Axial plane imaging

**Figure 4:**
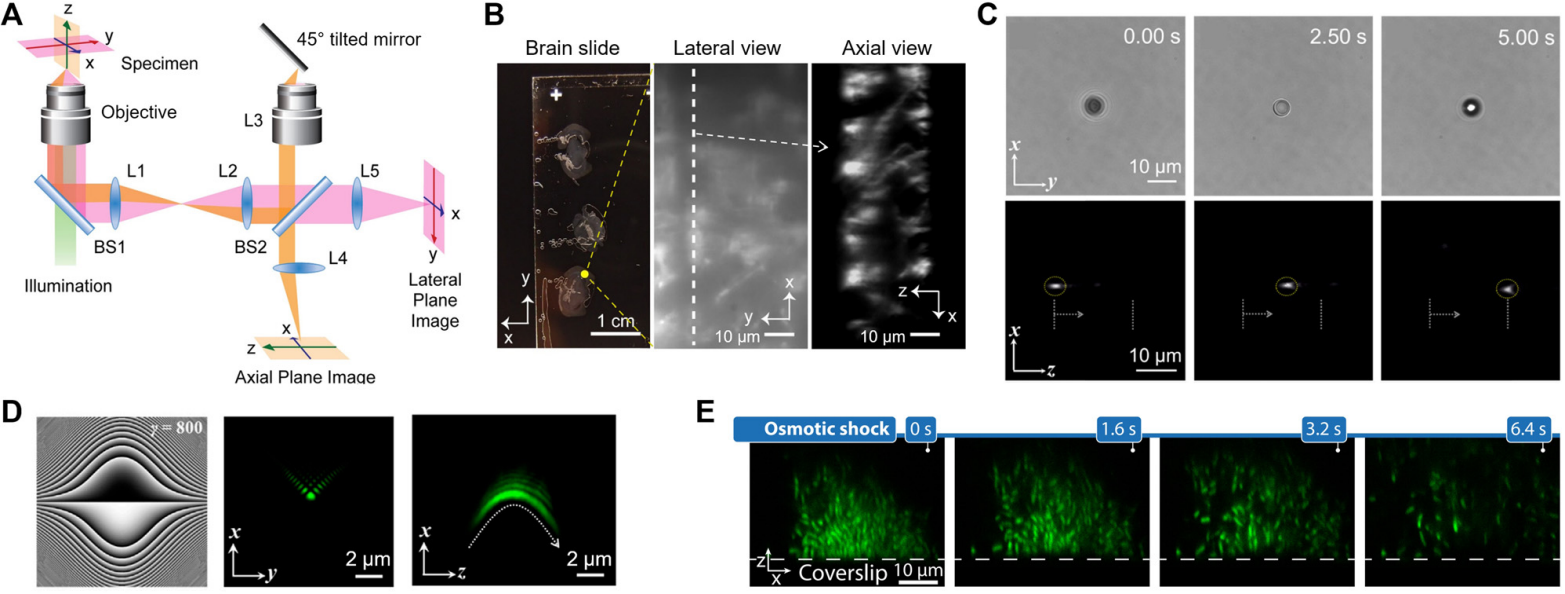
Applications of axial plane optical microscopy (APOM). (A) Schematic of APOM. BS: beam splitter, L: lens. (B) APOM imaging of mouse brain slices. (A, B) were adapted from [27] (CC BY-NC-ND 4.0). (C) Lateral (*xy*) and axial (*xz*) views of a polystyrene bead moving along the Bessel beam axis (*z*). Adapted with permission from [11] © The Optical Society. (D) Phase mask of the Weber beam and experimental lateral/axial images of the generated Weber beam. Adapted from [42] (CC BY). (E) Time-lapse axial plane imaging of *Vibrio cholerae* biofilms under osmotic shock. Adapted from [48] © The Optical Society.

Axial plane microscopy is an extreme case of widefield OPM when *α* = 90° and can be achieved with the single-objective layout in remote space with a 45° tilted mirror as illustrated in Figure 4A. Li et al. [27] demonstrated this large-angle imaging method, called axial plane optical microscopy (APOM). 2D fluorescence images of pine pollen grains and mouse brain slices along the axial plane were directly obtained with lightsheet illumination enabling optical sectioning, and y-stacks were acquired with sample stage scanning to obtain high-contrast 3D images. A portion of the fluorescent signal was used to simultaneously image the lateral plane, providing additional information about the sample. APOM accommodates typical tissue slice samples well due to its single-objective geometry in sample space and enables high signal-to-background imaging compared to conventional epi-illumination fluorescence microscopy (Figure 4B).

APOM has been shown to be suitable for optical trapping applications. When a microparticle is trapped by a non-diffracting beam such as a Bessel beam [40], the particle can move along the axial propagation direction of the trapping beam. Unlike scanning remote focusing microscopy [41], APOM can directly image the movement process of the particle through the axial plane in real time, as demonstrated in Figure 4C with a polystyrene bead trapped in the Bessel beam [11]. This direct observation made it easy to analyze the change in velocity of the trapped particle as it moves axially due to the change in net axial force. APOM can be useful not only to visualize particle transport through trapping beams, but also to study axial propagation of self-accelerating beams (Figure 4D) [42]. The APOM approach does not rely on special sample devices or geometrical requirements for sample preparation compared to other axial imaging methods [43–46]. APOM could also be applied in cellular mechanobiology for side-view imaging of cells undergoing deformation [47]. In addition, APOM can be converted to digital-scanning confocal lightsheet microscopy (DC-APOM), where the 1D lateral scan of the Gaussian or Bessel beam is synchronized with the rolling shutter readout of the scientific complementary metal-oxide-semiconductor (sCMOS) camera serving as a virtual confocal slit [48]. DC-APOM showed much reduced photobleaching compared to spinning disk confocal microscopy and was suitable for imaging the dynamics of biological samples occurring in a direction perpendicular to standard microscope coverslips or multi-well plates (Figure 4E).

### 3.2 Super-resolution imaging

**Figure 5:**
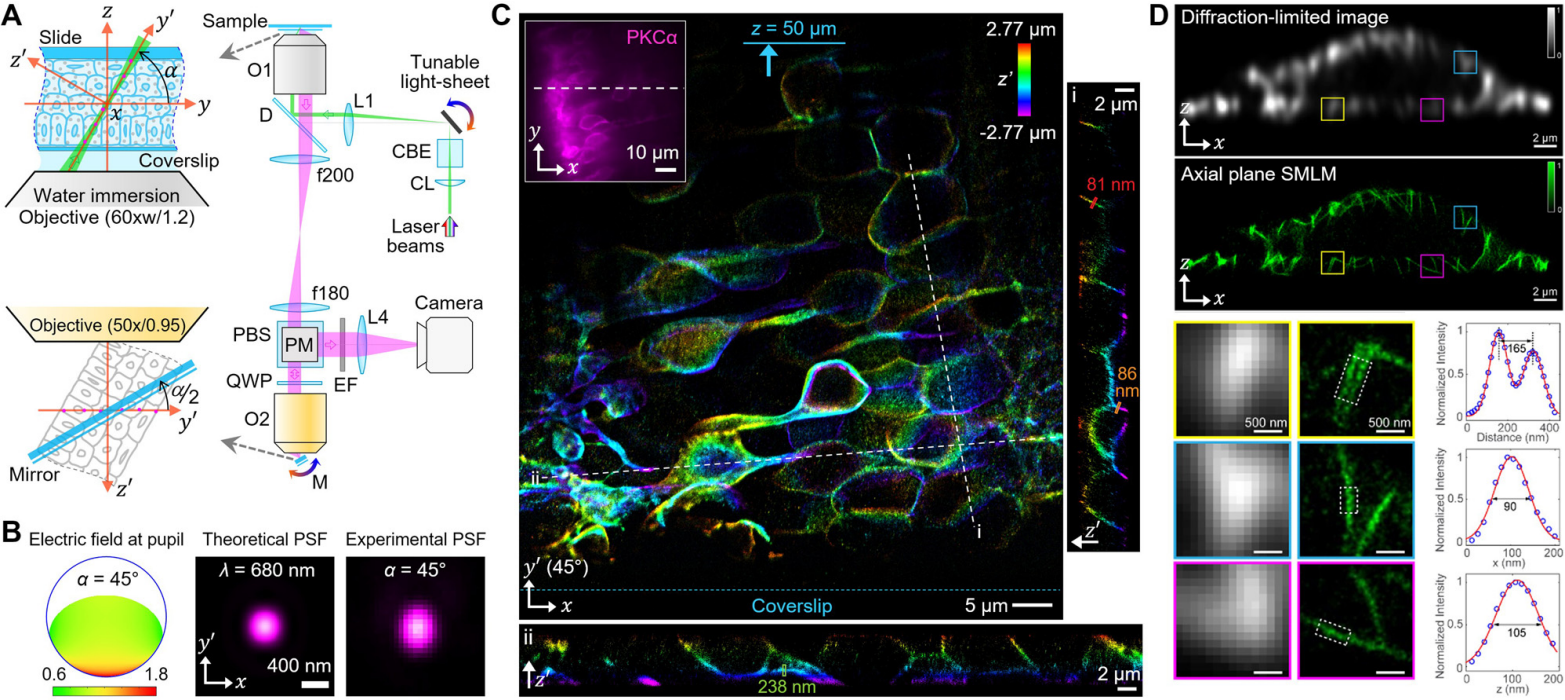
Single-molecule OPM imaging. (A) Schematic of oblique-plane SMLM (obSTORM). O: objective lens, D: dichroic beam splitter, L: lens, PBS: polarizing beam splitter, QWP: quarter-wave plate, PM: periscope mirror, M: mirror, EF: emission filter, CBE: cylindrical beam expansion, CL: cylindrical lens. (B) Theoretical pupil field and PSF at *α* = 45°, and measured PSF with dark-red beads. (C) Volumetric obSTORM image of mouse retina sections. The inset image shows the conventional lateral fluorescence image with the dashed line where the obSTORM image was taken. (A–C) were adapted from [12] © Springer Nature. (D) Comparison of diffraction-limited and SMLM images (*α* = 90°) of Alexa Fluor 647-labeled microtubules in COS-7 cells. Magnified view of three boxed areas and intensity profile of microtubules marked by dotted boxes. Adapted from [13] © The Optical Society.

Widefield OPM has been successfully combined with single molecule localization microscopy (SMLM) [49–51] for super-resolution imaging. Kim et al. [12] demonstrated oblique-plane lightsheet SMLM, named obSTORM, in which single molecule imaging is acquired along an oblique plane as illustrated in Figure 5A. The operating angle (*α*) of obSTORM can be easily changed between 45° and 90° by adjusting the tilt of the two mirrors, one in the illumination path for lightsheet illumination and the other in remote space for OPM, and these larger imaging angles are well-suited for thicker biological specimens and can expand SMLM applications beyond the single-cell level. OPM’s intrinsically elliptical PSF was improved to be nearly 2D isotropic at *α* = 45° in obSTORM by using the polarization component of the fluorescence signal that enhances the pupil field in the *y’* direction and using a remote objective with a larger NA than the sample objective (Figure 5B). Due to the single-objective layout in sample space, the obSTORM platform is fully compatible with standard tissue and small animal samples prepared for conventional fluorescence microscopy. Imaging performance was demonstrated primarily at *α* = 45° using a variety of specimens, from cells to *Caenorhabditis elegans* gonads, *Drosophila melanogaster* larvae, mouse retina and brain sections, and small fish. obSTORM with Alexa Fluor 647 dyes showed a localization precision of ∼18 nm for thin cells and ∼26 nm for tissues and an excellent imaging depth of up to 66 μm in mouse retina sections. 3D localization from the astigmatic PSF using a cylindrical lens [52] also worked well in obSTORM, enabling super-resolution volumetric imaging of tissue samples as shown in Figure 5C. B. Huang’s group also demonstrated oblique epi-illumination SPIM (eSPIM) operating at *α* = 30° with SMLM imaging capability [18]. eSPIM improved light collection efficiency at the third objective (water immersion) interfaced with the second objective (air) via a 3D-printed water chamber. This special index mismatch design resulted in an average single-molecule photon count of >2400 photons for Alexa Fluor 647 (∼30 % of the photon count in a 1.4 NA oil-immersion objective) and produced SMLM images of ∼50 nm wide microtubules in *Drosophila* S2 cells.

Axial plane SMLM (*α* = 90°) was demonstrated in detail by An et al. [13], which resolved nanoscale cellular structures along the axial direction without z-scanning. When using two 1.4-NA oil-immersion objectives for remote focusing, the FWHM size of the elliptical PSF was ∼860 nm in the *z* direction, and the PSF shape was well maintained over 20 μm even in aqueous specimens with refractive index mismatch. In 2D axial SMLM imaging of fixed COS-7 cells (Figure 5D), the averaged FWHM of microtubules labeled with Alexa Fluor 647 was ∼103 nm in the axial direction (axial localization precision: ∼44 nm), with no significant difference over 8 μm sample depth. The minimal background fluorescence with lightsheet illumination also made the technique compatible with DNA-PAINT (points accumulation for imaging in nanoscale topography) [53] and Exchange-PAINT [54] without photobleaching issues due to the infinite supply of single molecules. Therefore, axial plane SMLM has shown strong potential to be a valuable tool for studying axial cellular structures and processes in fixed and living samples.

**Figure 6:**
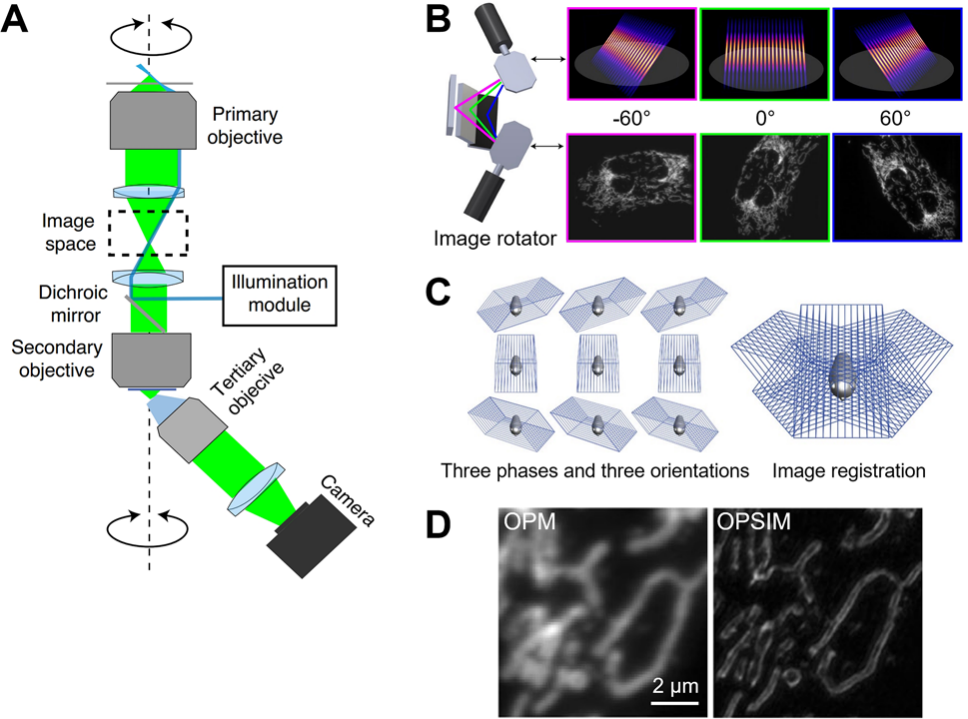
Super-resolution OPM with structured lightsheet illumination. Adapted from [14] with permission from Springer Nature. (A) Simplified schematic of OPSIM. (B) Optical rotator of lightsheet illumination and fluorescence detection optics for three azimuthal orientations. (C) OPM stack acquisition and image registration for SIM reconstruction. (D) Comparison of OPM and OPSIM images of mitochondria in U2OS cells.

Recently, Chen et al. [14] developed oblique plane SIM (OPSIM) that combines widefield OPM (*α* = 45°) and structured illumination microscopy (SIM [55]) for super-resolution cellular imaging as illustrated in Figure 6A. Realizing SIM illumination patterns at multiple azimuthal angles can be difficult with the conventional SPIM layout [2] since two additional illumination objectives will be required for each ±60° angle (thus a total of four objectives in sample space). OPSIM employs the single-objective lightsheet layout with an image rotator, consisting of a pair of galvanometric mirrors and three static mirrors, to azimuthally rotate the lightsheet and detection optics by 60° at millisecond time intervals (Figure 6B). A total of nine oblique stacks are acquired at one time point across three azimuthal orientations and three structured illumination phases and then computationally registered with each other in *xyz* Cartesian coordinates (Figure 6C), and 2D SIM reconstruction is performed for each *xy* layer. Compared to OPM, OPSIM showed superior imaging resolution as shown in Figure 6D. OPSIM exhibited an isotropic lateral resolution of <150 nm and an axial resolution of <500 nm and enabled volumetric imaging at ∼1 Hz with much reduced photobleaching and phototoxicity compared to conventional SIM.

### 3.3 High-speed 3D imaging

**Figure 7:**
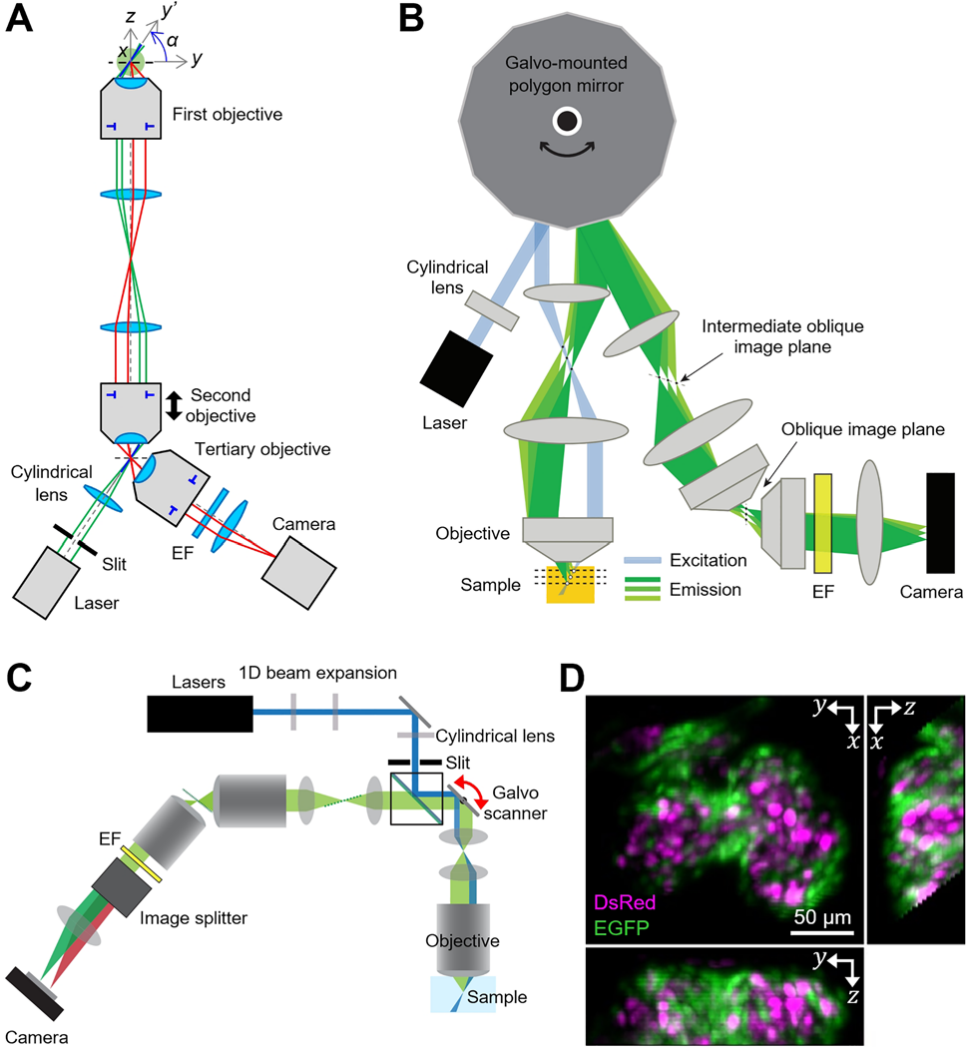
High-speed volumetric OPM. (A) Schematic of objective-scanning OPM. EF: emission filter. (B) Schematic of SCAPE system. Adapted with permission from [15]. (C) Schematic of SCAPE 2.0. Adapted with permission from [17]. (D) Maximum intensity projection images of zebrafish heart by SCAPE 2.0. Red blood cells and endothelial cells were stained with DsRed and EGFP, respectively. Adapted from [17] with permission from Springer Nature.

The most exciting advances in widefield OPM are towards high-speed 3D imaging. Rapid volumetric imaging in widefield OPM was initially achieved by C. Dunsby’s group through axial scanning of the second objective by piezoelectric objective scanner as illustrated in Figure 7A [37, 56]. As lightsheet illumination was provided through the second objective in remote space for optical sectioning, it was sufficient to move only the second objective for 3D imaging without sample agitation. This remote objective-scanning approach was much faster than conventional sample stage-scanning approaches [3, 27, 57], and the 3D image acquisition speed was determined by the scanning speed of the second objective and the frame rate of the camera. They demonstrated 3D imaging of calcium dynamics in rat cardiomyocytes using an electron-multiplying CCD (EMCCD) camera at 21 volumes per second (VPS) over a volume of 72.6 × 36.3 × 36.1 μm^3^ (128 × 64 × 20 voxels; 20 optical sections/volume) in 2011 [56]. In 2015, they also demonstrated two-color volumetric imaging of cardiac myocytes at 25 VPS with 960 × 200 × 20 voxels (20 sections/volume) using two high-speed sCMOS cameras [37].

In 2015, E. Hillman’s group proposed the concept of 1D optical scanning and descanning in widefield OPM for rapid volumetric imaging [15]. Their technique, called swept confocally-aligned planar excitation (SCAPE) microscopy, uses a polygonal scanning mirror to laterally sweep oblique lightsheet illumination at varying angles (*α* = 50–70°) across the specimen and conjugate this moving oblique plane to a stationary camera, thereby acquiring oblique stacks without scanning the specimen or objective lens (Figure 7B). SCAPE imaging speed was primarily limited by camera readout speed and fluorescence signal-to-noise ratio. Using an sCMOS camera (2404 FPS for 80 rows), they imaged the beating heart within freely moving transgenic *Drosophila* larvae at 20 VPS over 120 × 800 × 80 voxels (120 sections/volume; FOV: 430 × 1330 × 134 μm^3^; first objective NA: 0.95 or 1.0). In 2018, the 1D optical scanner was replaced with a one-axis galvanometer mirror that can keep the imaging angle (*α*) unchanged while scanning/descanning the specimen (Figure 7C), demonstrated by Hillman et al. [58] and Kumar et al. [16]. Rapid *in vivo* volumetric imaging by SCAPE microscopy was shown to be useful for characterizing proprioceptive system dynamics in crawling *Drosophila* larvae [59]. The volumetric imaging speed was significantly improved to >100 VPS on the SCAPE 2.0 platform [17] by using a high-speed intensified CMOS camera (pixel readout rate: ∼1.2 GHz) capable of fluorescence signal enhancement [60]. SCAPE 2.0 also featured several improvements, including more uniform oblique lightsheet illumination (*α* = 53°) using a Powell lens, flexible layout changes between upright/inverted/side-view configurations, and switching of a third microscope system for different resolutions (effective NA in the *y’* direction: 0.23–0.48) and magnifications (4.6×–67×). The fastest dual-color imaging rate demonstrated was 321 VPS, visualizing the zebrafish heartbeat (Figure 7D) over a volume of 197 × 293 × 78 μm^3^ (57 sections/volume; ∼6.21× magnification). The powerful potential of SCAPE for *in vivo* real-time histological imaging of living tissues was demonstrated with the benchtop and miniaturized MediSCAPE prototypes [19]. Gong et al. proposed a SCAPE system in which two 20×/1.0 NA water immersion objectives are immersed in a water tank in remote space [61]. Due to the higher cone angle of the tertiary objective than the air objectives used in SCAPE 2.0, this approach was claimed to improve imaging resolution and fluorescence collection efficiency. OPM and SCAPE technologies have been licensed to Leica Microsystems for commercialization [62–64].

Volumetric imaging of widefield OPM using optical scanning was also developed by other research groups around the same time. In 2017, H.S. Kwon’s group proposed a refractive 1D scanner that laterally shifts the optical axis by tilting a flat glass window [65]. The constant sweep angle (*α* = 67°) of this approach made the sampling resolution uniform and thus simplified post-image processing of the initial SCAPE. With scattering-robust lightsheet illumination using a 1D scanning two-photon Bessel beam, they demonstrated 3D imaging of pollen grains and mouse optic nerves at 0.4 VPS (limited by the camera used). In 2019, eSPIM [18] from B. Huang’s group used a 1D galvanometer scanner for 3D imaging (*α* = 30°) and a water immersion tertiary objective with a custom-made water container for high NA cellular imaging. The effective detection NA achieved was 1.06 (or 1.18 according to [38]) in the *y’* direction. The average FWHM of the PSF for *λ* = ∼525 nm was 316, 339, and 596 nm in the *x*, *y’*, and *z’* directions, respectively, when measured with Gaussian lightsheet illumination, and the PSF was made smaller with Bessel lightsheet illumination. With the sCMOS cameras running at 800 FPS for 640 × 256 pixels, the achievable imaging speeds were 0.5–2 VPS over a volume of ∼100 × 70 × 20 μm^3^ (magnification: 50×) or increased up to 15 VPS over a smaller volume (35 × 35 × 7 μm^3^; 34 sections/volume). They successfully demonstrated time-lapse parallel imaging of S2 cells in 96-well plates with drug treatment. In 2018, Y. Kozorovitskiy’s group introduced scanned oblique plane illumination (SOPI) microscopy [16], which uses one- and two-photon illumination from a cylindrically focused lightsheet with a constant sweep angle (*α* = 45°) by a galvanometer mirror. One-photon volumetric calcium imaging of *in vivo* zebrafish larvae was demonstrated at 10 VPS with an sCMOS camera over a volume of 850 × 300 × 50 μm^3^ (10 sections/volume; effective NA: 0.34 in the *y’* direction; magnification: ∼15×). Compared to one-photon illumination, two-photon illumination improved imaging resolution and reduced shadow artifacts in highly scattering samples, but slowed down imaging rates and reduced illumination coverage in the direction of sheet propagation. The illumination was replaced by 1D scanning of a laser beam [66] for improved lightsheets in SOPI 2.0 [67] in 2019, where they demonstrated large-scale volumetric imaging (>1 mm^3^) of mouse brain sections at cellular resolution by stitching multiple tiles obtained by stepping the specimen. The geometrical optics behind the tilt-invariant lateral scan in volumetric OPM was also studied in detail [68].

**Figure 8:**
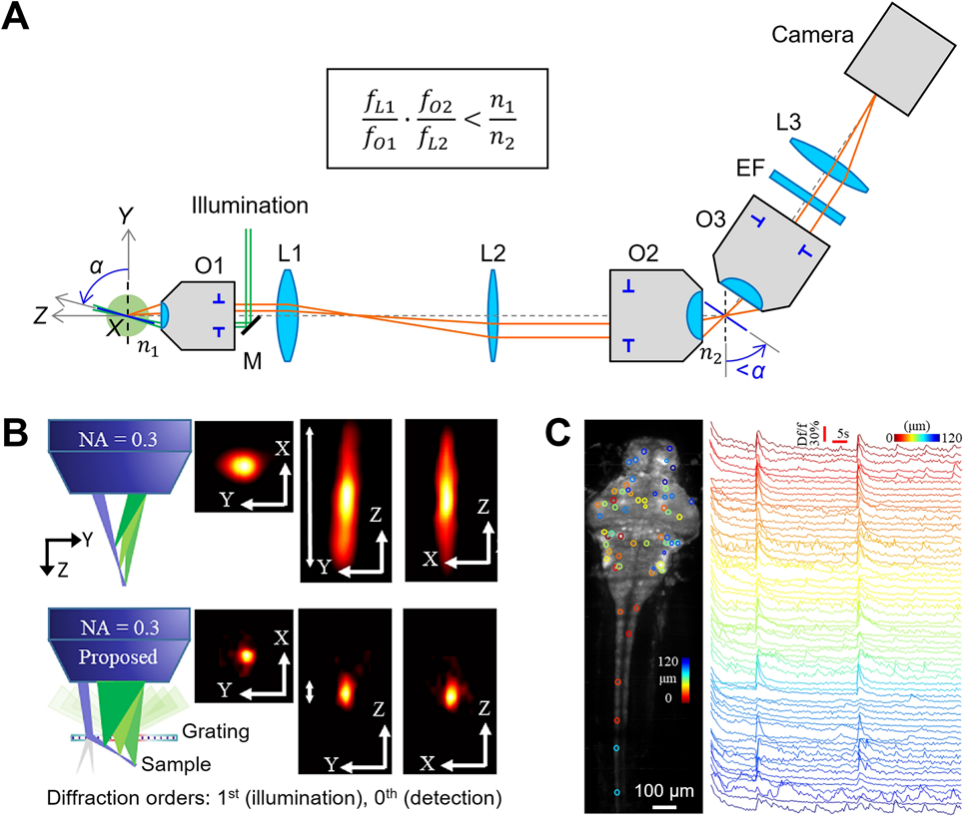
Mesoscopic OPM. (A) Simplified schematic diagram of mesoscopic OPM. O: objective lens, L: lens, M: mirror, EF: emission filter, *f*: focal length, *n*: refractive index. (B) Resolution improvement with diffractive lightsheet illumination. (C) Volumetric calcium imaging of zebrafish larvae. Color indicates sample depth. (B, C) were adapted from [71] © Optica Publishing Group.

Volumetric OPM was also developed by J. Yi’s group. In 2017, they introduced oblique scanning laser microscopy (OSLM) [69], combining widefield OPM (*α* = ∼64°) and optical coherence tomography (OCT) for simultaneous molecular and structural volumetric imaging at cellular resolution. One of the two galvanometer scanning mirrors on the illumination side was used for A-line scanning (50 kHz) for OCT and lightsheet generation for OPM. The other scanner was used for slow raster scanning for volumetric imaging and was synchronized with a separate descanner for fluorescence detection on a CCD camera. Imaging demonstrations included *ex vivo* 3D fluorescence and OCT imaging of mouse retinal vasculature. Also, they developed mesoscopic OPM [21] for a large FOV of up to ∼6 × 5 × 0.7 mm^3^ by using lower NA objectives (first objective NA: 0.3; *α* = ∼72°) and implementing OPM based on the Scheimpflug condition [70] rather than the remote focusing condition. As illustrated in Figure 8A, this design makes the lateral magnification from sample space to remote space smaller than *n*_1_/*n*_2_ (or one in air), which reduces the imaging angle in remote space to smaller than *α* (in sample space) and consequently improves the light collection efficiency in the third objective. Moreover, by placing a transmission grating between the first objective and the specimen, they made the angle of the oblique lightsheet smaller than allowed by the 0.3-NA objective, leading to a 6-fold improvement in axial imaging resolution (∼6 μm) in mesoscopic OPM in terms of the system PSF (Figure 8B) [71]. As shown in Figure 8C, they demonstrated *in vivo* volumetric imaging of whole zebrafish larvae, recording neuronal activity at 2 VPS (125 sections/volume) with a spatial resolution of 2.5 × 3 × 6 μm^3^.

J. Yi’s group also demonstrated the great potential of volumetric OPM for application in ophthalmic research. In 2018, they developed oblique scanning laser ophthalmoscopy (oSLO) [72, 73], which uses the optics of the subject’s eye as the first objective in OPM (*α* = 75°). *In vivo* volumetric fluorescein angiography (vFA) of the mouse retina was performed at 2 VPS (100 sections/volume) with a CMOS camera [74]. They also showed the potential of oSLO for *in vivo* human retinal imaging with an FOV of ∼3 × 6 × 0.8 mm^3^ at the lateral and axial resolutions of 7 μm and 41 μm, respectively [75]. Recently, they reported confocal oSLO (CoSLO) based on confocal slit detection to improve image contrast and resolution compared to conventional SLOs and demonstrated *in vivo* volumetric human retinal imaging at ∼0.2 VPS [76].

### 3.4 Other technological advances and applications

**Figure 9:**
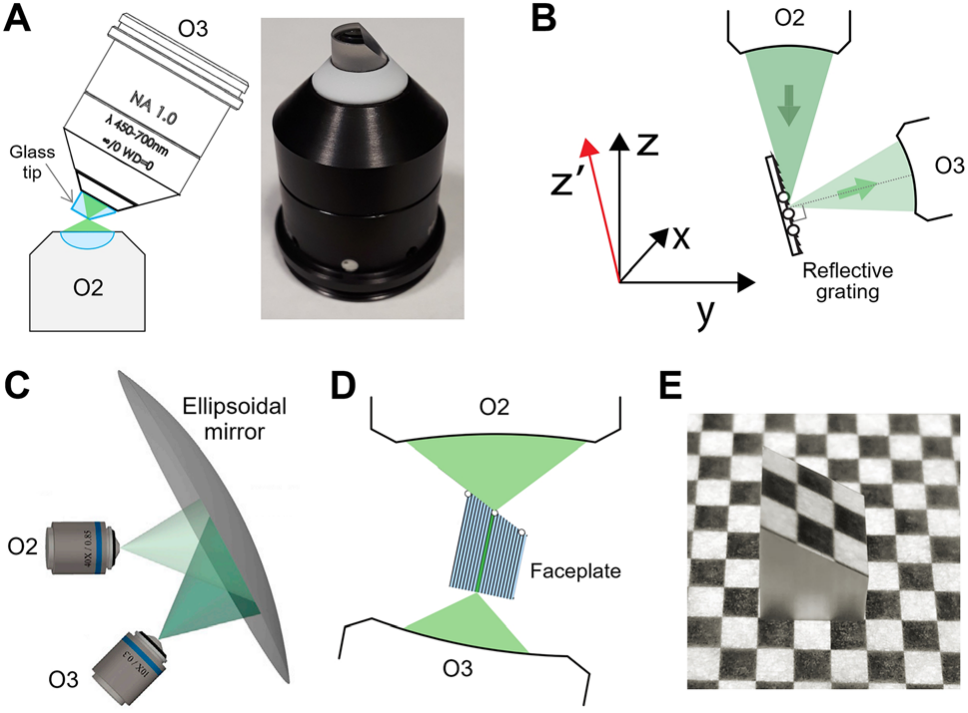
Various optical elements used in OPM’s remote space. O2: secondary objective, O3: tertiary objective. (A) Glass-tipped tertiary objective (AMS-AGY v1.0). Adapted from [77] (CC BY 4.0). (B) Reflective grating. Adapted from [20] © The Optical Society. (C) Ellipsoidal mirror. Adapted from [80] (CC BY). (D) Fiber optics faceplate. (E) Photograph of the custom faceplate placed on a checkerboard pattern. (D, E) were adapted with permission from [24].

Beyond eSPIM’s 3D-printed water chamber in remote space [18], widefield OPM technology has driven the development of custom objectives. A. Millett-Sikking and A.G. York in Calico Life Sciences invented a bespoke glass-tipped objective (AMS-AGY v1.0, NA: 1.0, focal length: 5 mm) for use as a tertiary objective in OPM compatible with *α* = 0–45° [77]. This special lens helps to achieve high effective NA in the *y’* direction through its air–glass interface at the focal plane by minimizing pupil loss (Figure 9A). In 2020, using the AMS-AGY v1.0 objective, R. Fiolka’s group constructed a volumetric OPM (*α* = 30°) with a large lateral FOV of 180 × 180 μm^2^ and an FWHM resolution of 299 nm (*x*), 336 nm (*y*), and 731 nm (*z*) from green fluorescent bead images [25]. Imaging resolution was further improved by ∼30 % with PSF deconvolution, and they demonstrated volumetric imaging of subcellular dynamics at up to 14 VPS. Using the longer focal length (9 mm) version of AMS-AGY objective (v2.0, NA: 1.0), Yang et al. [38] developed DaXi (effective NA: 0.97 in the *y’* direction), a 14.8× low-magnification OPM system for a larger FOV in 2022. DaXi achieved dual-view imaging (*α* = ±45°) using an optical image flipper with two galvanometer scanners. This approach can be much faster than C. Dunsby’s dual-view OPM (dOPM) in 2020 [78], which is based on mechanical flipping by laterally moving a pair of ±22.5° tilted remote mirrors. Both groups applied PSF deconvolution and image fusion of the two views to improve image resolution and quality. Dual illumination in two orthogonal directions also improved volumetric coverage and suppressed scattering and shadowing artifacts. The usefulness of DaXi for developmental biology studies was demonstrated with long-term volumetric imaging of large samples including *Drosophila* egg chambers and zebrafish larvae. The FOV of OPM with the AMS-AGY 2.0 objective was extended several times by R. Fiolka’s group using a dual-axis scanning unit for optical tiling that is much faster than conventional mechanical tiling by specimen scanning [23]. The group also developed a multi-angle projection imaging method based on a shear-scan unit that, when applied to the OPM system, can eliminate the computational shear correction of OPM image stacks [79]. This method can also faciliate sample navigation and potentially speed up the process of volumetric imaging significantly. AMS-AGY objectives are currently available from Applied Scientific Instrumentation, Inc. (ASI).

Various optical designs in remote space have been proposed to improve the OPM’s imaging performance. In diffractive OPM (*α* = 79.1°) [20], it was possible to construct a volumetric OPM system with lower NA objectives (NA: 0.28) by redirecting the propagation of the intermediate image in remote space through a reflective diffraction grating. As illustrated in Figure 9B, the blazed grating surface placed on the intermediate oblique plane diffracted the first-order light into the 4× tertiary objective with 43 % efficiency for large volumetric OPM imaging. The diffractive OPM had an accessible FOV of 3.3 × 3.0 × 1.0 mm^3^ with an FWHM resolution of 2.6 × 3.1 × 37.4 μm^3^ and demonstrated its practicality with time-lapse calcium imaging of the whole zebrafish brain at 0.8 VPS. An OPM system similar to the diffractive OPM was also presented in Ref. [22]. On the other hand, Liu et al. [80] proposed using an ellipsoidal mirror (EM) to relieve the mechanical constraints of the two objective configurations in conventional remote focusing (Figure 9C). As the EM conjugates the intermediate oblique plane from the second objective to the focal plane of the tertiary objective, the two objective lenses can be positioned apart without pupil loss in the *y’* direction. Therefore, compared to the PSF of conventional widefield OPM, the selection of the tertiary objective is flexible and a brighter and more isotropic PSF can be obtained even at higher image tilts such as *α* > 40°. They showed in optical simulations that this concept also works well for OPM with larger FOVs if the higher-order terms of the EM surface are optimally designed. Another interesting idea from Hoffmann et al. [24] is image transfer OPM (ITOPM, *α* = 69° or 57°) which uses an angled fiber optic faceplate (FP, apex angle: 35°) in remote space as shown in Figure 9D. The FP consists of an array of multimode fibers (fiber diameter: 2.5 μm, NA: 1.0) such that the intermediate image formed at one end of the FP is transferred to the other end (Figure 9E). Therefore, it allows the use of low magnification/NA objectives for large FOV OPM without sacrificing light collection efficiency and effective detection NA. ITOPM exhibited an FOV of 2.4 × 2.1 × 1 mm^3^ with an average FWHM resolution of 2.7 × 3.1 × 21 μm^3^ measured from fluorescent beads, suitable for brain-wide calcium imaging of adult *Danionella* fish at 1 VPS (318 sections/volume) at cellular resolution.

**Figure 10:**
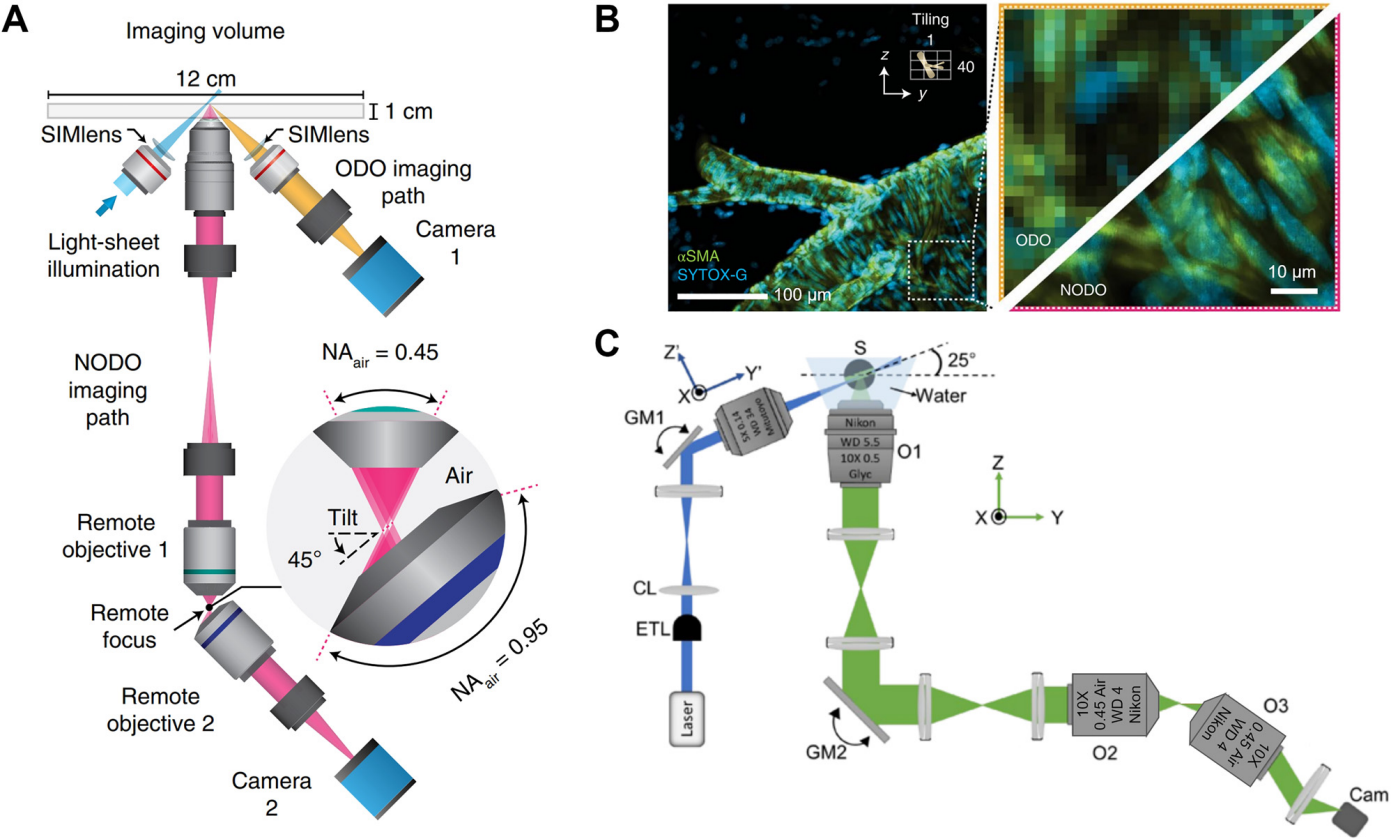
Open-top dual-objective OPM. (A) Hybrid open-top platform of NODO (non-orthogonal dual-objective) OPM and ODO (orthogonal dual-objective) lightsheet microscope. SIMlens: solid-immersion meniscus lens. (B) Two-color ODO imaging of a cleared mouse brain labeled with arterial (αSMA) and nuclear (SYTOX-G) stains and magnified views of ODO and NODO images in subregions. (A, B) were adapted from [83] with permission from Springer Nature. (C) MesOPM with optical scanning for volumetric imaging. GM: galvanometer mirror, S: sample, CL: cylindrical lens, ETL: electrically tunable lens, O: objective. Cam: camera. Adapted from [84] © Optica Publishing Group.

An OPM system using dual objectives in sample space was demonstrated. In a performance analysis between different architectures of open-top lightsheet microscopes [81, 82], J.T.C. Liu’s group found that for an NA of the first objective within 0.5–0.8, a non-orthogonal dual-objective (NODO) configuration in sample space can be advantageous in terms of imaging resolution/contrast, FOV and working distance, along with flexibility in optical design, compared to the single-objective configuration [26]. As schematically shown in Figure 10A, the group demonstrated a hybrid open-top platform of NODO OPM (*α* = 45°; effective NA: ∼0.45) and conventional orthogonal dual-objective (ODO) lightsheet microscopy (objective NA: 0.1) for versatile multi-scale volumetric imaging in cleared tissue applications [83]. The imaging system was fully compatible with cleared tissues with different geometries and refractive indices (Figure 10B), offering a lateral resolution of ∼0.5 μm at an imaging speed of ∼5 mm^3^ per hour in NODO mode or a lateral resolution of ∼10 μm at an imaging speed of 10 cm^3^ per hour in ODO mode. Recently, Singh et al. [84] also developed open-top and dual-objective OPM with a large FOV at cellular resolution, named mesoscopic OPM (MesOPM) as illustrated in Figure 10C. Since the lightsheet illumination was provided separately at a small angle (*α* = 25°), the axial elongation of the system PSF was minimized to ∼5 μm for the low NA OPM system (effective NA: 0.23). Moreover, two galvanometer scanners (one for sweeping oblique illumination and the other for fluorescence descan) were used synchronously to achieve fast volumetric OPM without sample agitation. They demonstrated MesOPM imaging of freely moving sea anemone (*Nematostella vectensis*) over a volume of 1.56 × 1.56 × 0.25 mm^3^ at 0.5 VPS with an FWHM resolution of 1.6 × 2.8 × 5.3 μm^3^.

**Figure 11:**
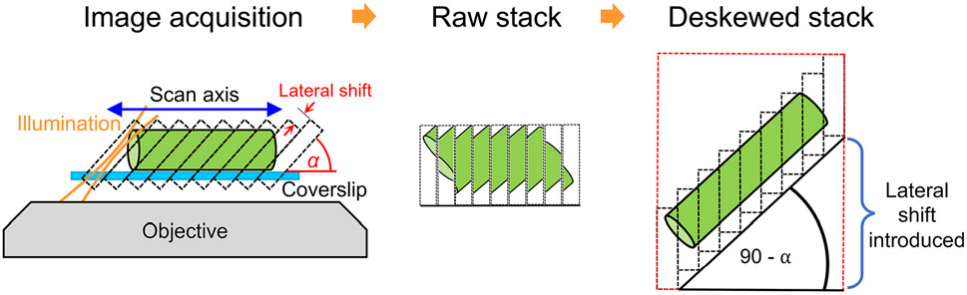
De-skewing of OPM image stacks for correct 3D visualization. The OPM image of the sample (here the green cylinder) is distorted in the raw image stack, but correctly visualized in the de-skewed stack where the lateral shift between images is corrected. Adapted from [86] (CC BY 4.0).

OPM-related software has also been developed. Recently, C. Dunsby’s group developed two MATLAB-based software packages (Catalogue Generator and Doublet Selector) to automatically design a 4f system based on commercial lens combinations, connecting the two objectives with the correct magnification for remote focusing [85]. Lamb et al. [86] developed a live visualization software package (open source) built on a Python-Micromanager interface that properly transforms the oblique stack of OPM imaging data on a graphics processing unit (GPU). As illustrated in Figure 11, the software displays correctly deskewed OPM images at a rate of several Hz, making real-time operation of OPM during sample navigation more intuitive and user-friendly.

From an application point of view, OPM may be well-suited for visualizing microfluidic environments. Although at an early application stage, lightsheet microscopy is increasingly being applied as an imaging platform for lab-on-a-chip (LOC) devices because of its lower phototoxicity and faster imaging speed than confocal microscopy [87]. However, in a single-objective SPIM configuration, fluidic chips require a 45° reflector on the sidewall [88] or near the fluid channel [89, 90], which adds additional effort in chip design and fabrication. Dual-objective SPIM eliminates the sidewall requirement, but the chip may have potential dimensional constraints if the objective’s working distance is not long enough [91]. Image quality can also be degraded by optical aberrations induced in illumination and detection through the 45° interface especially when using high NA objectives. Widefield OPM may mitigate these potential issues. In fact, Ugawa and Ota [92] recently demonstrated that OPM can be successfully applied to optofluidic flow cytometry for volumetric imaging of cells in parallel at subcellular resolution, allowing cell counting at a high throughput of 3220 cells/s. In addition, McKay et al. [93] showed scattering-contrast OPM (sOPM) as a promising new imaging tool for *in vivo* blood analysis by demonstrating label-free imaging of human blood cells flowing in capillaries. Widefield OPM will also soon find applications in soft matter studies, such as capillary wave dynamics and droplet coalescence in colloidal fluid systems [94].

## 4 Oblique plane imaging by scanning microscopy

**Figure 12:**
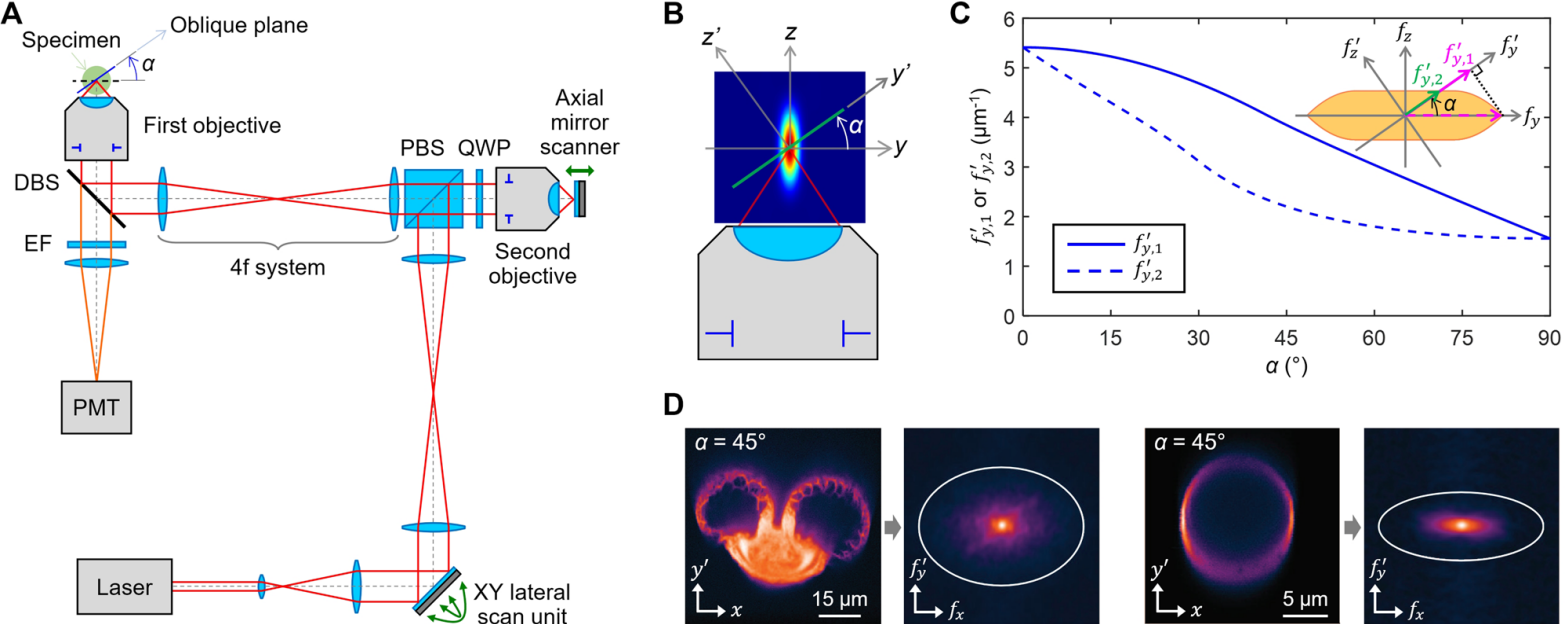
Oblique plane imaging by scanning microscopy. (A) Schematic of remote focusing-based scanning microscopy. DBS: dichroic beam splitter, EF: emission filter, PMT: photo-multiplier tube, PBS: polarizing beam splitter, QWP: quarter-wave plate. (B) Schematic of the PSF of remote focusing microscopy related to oblique plane imaging with angle *α*. (C) Spatial cutoff frequency over *α* for 1.15 NA (water immersion) at *λ* = 850 nm. The inset (top right) shows spatial frequency support for OTF. The two cutoff frequencies are *f’*_*y,*1_ (including projections of spatial frequencies out of the *xy*’ plane) and *f’*_*y,*2_ (the largest in-plane frequency). (D) Experimental fluorescence images (1.15 NA, water immersion) and Fourier frequency spectra of a pollen grain and a spherical shell. Adapted with permission from [105] © The Optical Society.

In addition to widefield OPM, oblique plane imaging has been demonstrated with remote focusing-based scanning microscopy. The remote focusing technique developed by T. Wilson’s group originally aimed to improve the axial scan rate of scanning microscopy by axially sweeping a small remote mirror mounted on a stage rather than conventional axial scanning driven by an objective lens or sample stage [5]. By using a pair of galvanometer motors instead of piezoelectric (PZT) stages, the axial scan rate was further accelerated with a bandwidth of ∼3.5 kHz [95]. The fast axial scans based on remote focusing work well for high NA systems without the negative effects of optical aberrations, as opposed to axial scans with electrically tunable lenses [96–98]. As illustrated in Figure 12A, remote focusing scanning microscopy consists of conventional lateral scan units (e.g., galvanometer scanners) and an axial scan unit (i.e., a small mirror in remote space), allowing fast 2D raster scans on any oblique plane [95]. Two-photon imaging of pollen grains captured at 45° and 90° angles was demonstrated using an “oblique-plane scanning multiphoton microscope” [99]. Two oblique plane images (*α* = ±45°) without conventional z-stack acquisition were also directly obtained by Botcherby et al. [100] to rapidly extract myocyte orientation and sarcomere length at the cell level in living Langendorff perfused rat hearts. Oblique plane imaging by remote focusing microscopy was also applied to simultaneously image more than 100 adjacent cells in the retinotopic plane of the *Droshophila* visual system at ∼15 frames per second (FPS) [101]. The 10-fold increase in imaging throughput made remote focusing microscopy suitable for high-throughput functional studies of the visual system on complex stimuli. In addition, remote focusing was applied to slit-scanning confocal microscopy to enable real-time meridional plane (*xz*) imaging (*α* = 90°), where imaging speed was limited by charge-coupled device (CCD) cameras [102]. Two-photon fluorescence microscopy with remote focusing enabled axial scan rates of 100 Hz to visualize the switching dynamics of liquid crystals (LCs) across the LC layer [103]. Besides, remote focusing was used to substantially extend the depth of field (DOF) in real time in Nipkow disc confocal microscopy (DOF: ∼20 μm at 1.4 NA) [104] and widefield fluorescence microscopy (DOF: ∼150 μm at 0.6 NA) [34].

The imaging resolution of remote focusing microscopy associated with oblique plane imaging is also anisotropic, but has a different origin from widefield OPM. This is evident as shown in Figure 12B when considering the oblique cross-section of a typical “ellipsoidal” 3D PSF with an axially elongated shape [30]. The size of the PSF in the *y’* direction is always greater than the *x* (or *y*) direction and becomes as large as the axial PSF when *α* = 90°. Therefore, it can be predicted that the imaging resolution does not change in the *x* direction but degrades in the *y’* direction as *α* increases. This non-isotropic behavior was studied in terms of the passband of the 2D OTF, which was derived geometrically by projecting the 3D OTF along the frequency direction of the *z’* coordinate perpendicular to the oblique plane (Figure 12C) [105]. It was found that the cutoff frequency is constant in the *x* direction and decreases in the *y’* direction with increasing *α*, meaning that the imaging resolution is indeed worse in the *y’* direction. This result was also confirmed experimentally by two-photon imaging of pollen grains and spherical shells along a 45° oblique plane (Figure 12D). It is also interesting to note that the optical sectioning thickness is estimated to become thinner as α increases because the PSF along the *z’* direction becomes smaller [99].

**Figure 13:**
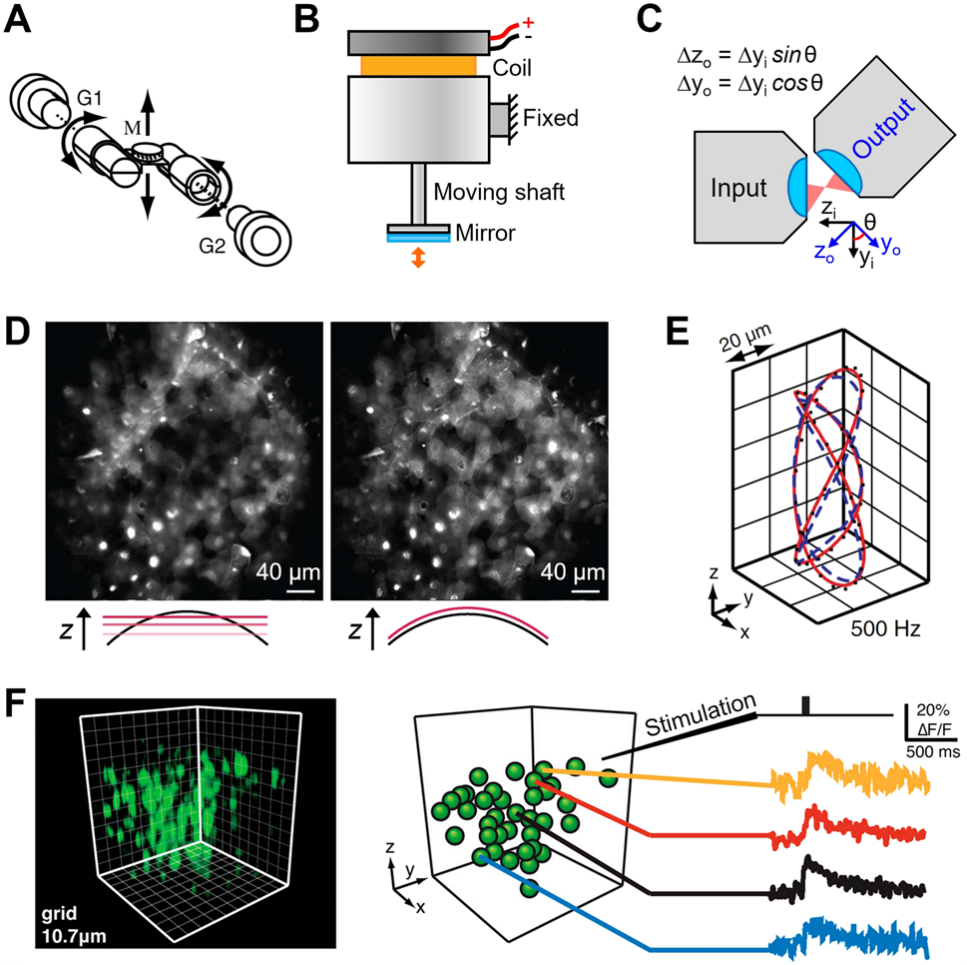
Advances in scanning remote focusing microscopy. (A) Custom-built axial scanner with a pair of galvanometer motors (G1, G2) and mirror M. (B) Voice coil motor with mirror. (C) Fast axial scan with pupil plane-actuated remote focusing. An optical lateral scan through the “input” objective is associated with an axial scan for the “output” objective. (D) Superficial epithelium imaging of mouse cornea. Projected image of 10 z-sections over 20-μm depth (left) and image of a single curved section (right). Adapted with permission from [114] © The Optical Society. (E) Fast 3D arbitrary line scanning. Blue and red lines represent the trajectories before and after calibration of the galvanometer response. (F) Functional 3D imaging of cortical neurons. (A, E, F) were adapted with permission from [106].

Remarkable advances have recently been made in scanning remote focusing microscopy. In addition to the custom-built axial scanner made with galvanometer motors as illustrated in Figure 13A [95, 106], several axial scanning methods have been proposed. As illustrated in Figure 13B, a voice coil motor was used to drive the small mirror in remote space at speeds comparable to PZT stages (several hundred Hz), but with a much longer range of motion [107]. Converting conventional lateral optical scanning to axial refocusing via a tilted flat or micro-staircase remote mirror allowed much faster axial scanning because the remote mirror did not need to be moved axially, and 12 kHz axial scan rates were demonstrated with lateral galvanometer scanning [108]. Fast axial scans without using the remote mirror were also made possible by “pupil plane actuated remote focusing” [109] where one-dimensional (1D) lateral scan through the angled objective produced an axial beam scan in sample space (Figure 13C). This approach enabled axial scans as fast as lateral scans by galvanometer motors and eliminated potential damage to the remote mirror by the high-power focus of the pulse laser used in multi-photon microscopy. Scanning remote focusing microscopy has also been applied to other microscopy modalities to avoid sample agitation, including spinning-disk confocal microscopy for volumetric calcium imaging of larvae [110] and interferometric scattering microscopy for long range axial tracking of nanoscale objects [111]. Another application includes dual-plane two- and three-photon microscopy [112, 113], where remote focusing and signal demultiplexing were utilized for *in vivo* calcium imaging of the mouse cortex.

In addition to oblique plane scanning, remote focusing microscopy can be used to scan curved surfaces or arbitrary 3D lines in general. As shown in Figure 13D, Colon and Lim [114] visualized a curved region of the corneal epithelium of the mouse eye in a single frame by driving the lateral and axial scan units with appropriate synchronization to form the desired imaging surface for ophthalmic imaging. The fast and arbitrary 3D line scanning capability over 100 μm in the *z* direction at unprecedented rates of 0.3–1 kHz (Figure 13E) also enabled high-speed functional calcium imaging of groups of cortical neurons simultaneously through a sample depth of ∼60 μm at 1 kHz acquisition rate (Figure 13F) [106].

## 5 Conclusions

OPM’s strategy for imaging along an inclined plane is unconventional because microscope objectives are designed to capture object information at the focal plane. Nonetheless, OPM can produce high-quality oblique plane images without the traditional z-stack acquisition that is slow and mechanically perturbs the specimen. The diffraction-limited performance in OPM is achieved using the optical technique of remote focusing that conjugates the pupils of the two objectives to eliminate aberrations for out-of-focus points on the oblique plane. OPM enables high-contrast 3D imaging with oblique lightsheet illumination, but at larger imaging tilts imaging resolution can be degraded due to pupil loss.

There have been many technical innovations made in OPM in recent years. Unlike the dual-objective geometry of conventional lightsheet microscopy, single-objective lightsheet OPM is fully applicable to standard microscopy samples such as laterally wide tissue sections and small animals. Moreover, sweeping an oblique image plane laterally across the specimen using an optical scanner, such as a galvanometer scanner, allows volumetric imaging without sample agitation at unprecedented speeds limited only by either camera readout rates or fluorescence signal-to-noise ratio. OPM-based volumetric imaging is much less susceptible to photodamage and phototoxicity compared to widely used confocal laser scanning microscopy, making it useful for long-term time-lapse observations of living biological samples. Widefield OPM can also be combined with existing super-resolution technologies, such as SMLM and SIM, to achieve super-resolution OPM. It has also been shown that the imaging performance of OPM, such as imaging resolution, signal brightness and FOV, can be improved by utilizing special optical elements including diffraction gratings, ellipsoidal mirrors, fiber optic faceplates, and customized glass-tipped objectives. Further technological development of OPM is expected from several perspectives. In the optics point of view, lightsheet illumination based on either two-photon excitation or non-diffracting beams will be further designed for deeper imaging in thicker specimens. The tertiary objective with higher NA and/or higher index glass tips could be developed for maximum effective NA in OPMs with higher imaging angles (*α*). An entirely new type of high NA objective lens that satisfies a stigmatic condition along an oblique plane may be available in the future. Moreover, continued advancements in camera technology and fluorescent probe technology will continue to improve OPM’s imaging speed and/or FOV. Despite unprecedented 3D imaging speeds, designing a system capable of easily switching imaging magnifications in one OPM system can be challenging due to the use of remote focusing with two or three objectives in an OPM set. It would be interesting to see how this design issue is addressed in future development. From a software point of view, researchers will develop real-time visualization of OPM data with deconvolution and fusion, large-capacity data compression for data transfer/storage, deep learning-based image enhancement and style transfer. There will also be application-driven OPM development where OPM systems are combined for multimodal imaging.

OPM offers an advanced volumetric microscope platform suitable for a variety of imaging applications. Indeed, the high spatiotemporal resolution of OPM has been proven optimal for fundamental biological/medical research at all levels of the system, from single cells to model organisms and small animals. For example, volumetric OPM can provide *in vivo* 3D calcium imaging to study neuronal dynamics in the brain of mice, *Drosophila* larvae, or zebrafish. Compared to the existing calcium imaging techniques that are limited to 2D imaging or operate in 3D on a limited number of neurons, OPM can better visualize the interactions between a much large number of neurons. OPM has also been shown to be effective for monitoring real-time drug treatment in single cells at subcellular resolution, which is expected to soon expand to studies using 3D cell culture models for more accurate testing. Additionally, the studies presented in this review demonstrated that OPM can be utilized for high-throughput flow cytometry and clinical diagnostics in the fields of hematology, histology, and endoscopy. However, it remains to be seen whether OPM will simply augment clinical diagnostics or will entirely replace traditional imaging approaches as a game changer. Other promising clinical applications of OPM include ophthalmic imaging combined with OCT angiography for clinical treatment of retinal conditions and basic vision science. In this application, the OPM system is being applied to the actual human eye beyond a simple feasibility study. In addition to the aforementioned fields, other fields of research, including but not limited to microfluidic devices and soft matter, are likely to emerge as new imaging applications for OPM in the near future. Therefore, OPM will be an important imaging tool for new scientific discoveries that are impossible or limited with conventional microscopy techniques.
